# Digging into the lesser-known aspects of CRISPR biology

**DOI:** 10.1007/s10123-021-00208-7

**Published:** 2021-09-06

**Authors:** Noemí M. Guzmán, Belén Esquerra-Ruvira, Francisco J. M. Mojica

**Affiliations:** 1 Dpto. Fisiología, Genética y Microbiología, Universidad de Alicante, Alicante, Spain; 2 Instituto Multidisciplinar para el Estudio del Medio, Universidad de Alicante, Alicante, Spain

**Keywords:** CRISPR, Cas proteins, Adaptive immunity, RNA-guided transposition, Non-canonical CRISPR roles, CRISPR regulation

## Abstract

A long time has passed since regularly interspaced DNA repeats were discovered in prokaryotes. Today, those enigmatic repetitive elements termed clustered regularly interspaced short palindromic repeats (CRISPR) are acknowledged as an emblematic part of multicomponent CRISPR-Cas (CRISPR associated) systems. These systems are involved in a variety of roles in bacteria and archaea, notably, that of conferring protection against transmissible genetic elements through an adaptive immune-like response. This review summarises the present knowledge on the diversity, molecular mechanisms and biology of CRISPR-Cas. We pay special attention to the most recent findings related to the determinants and consequences of CRISPR-Cas activity. Research on the basic features of these systems illustrates how instrumental the study of prokaryotes is for understanding biology in general, ultimately providing valuable tools for diverse fields and fuelling research beyond the mainstream.

## Introduction

The discovery of an RNA-based interference-like mechanism in prokaryotes (Mojica et al. 2005; Makarova et al. 2006), analogous to the adaptive immune system that operates in vertebrates, represented an unanticipated breakthrough in microbiology and immunology. Barrangou and collaborators (Barrangou et al. 2007) validated, in the lactic acid bacterium *Streptococcus thermophilus*, previous proposals relating clustered regularly interspaced short palindromic repeats (CRISPR) and Cas (CRISPR associated) proteins (Mojica et al. 2000; Jansen et al. 2002) to defence against invasive genetic elements (Mojica et al. 2005; Pourcel et al. 2005). These initial hypotheses were based on the analysis of CRISPR arrays in many *Yersinia pestis* genomes (Pourcel et al. 2005) and representative strains of the main taxonomic groups of archaea and bacteria (Mojica et al. 2005). The comparison of the CRISPR regions with sequences available in nucleotide databases revealed that repeat intervening *spacers* matched other sequences (later termed protospacers; Deveau et al. 2008) in mobile genetic elements related to the spacer-carrier strain. Moreover, the presence of a given spacer seemed to be incompatible with the co-occurrence in the cell of perfectly matching protospacers, suggesting the existence in prokaryotes of a CRISPR-based adaptable mechanism of protection (Mojica et al. 2005). Consequently, it was proposed that the immune memory relied on the integration of invading nucleic acids within the CRISPR loci. Indeed, we know now that sequences of foreign origin, either from RNA (Silas et al. 2016) or DNA (Barrangou et al. 2007), can be incorporated into CRISPR arrays during the infection process, resulting in new spacers framed by repeat units. Spacer acquisition is the first step of the CRISPR-Cas mechanism, named ‘adaptation’ or ‘immunisation’. The second stage is referred to as ‘expression’ or ‘CRISPR RNA (*crRNA*) biogenesis’ and the last one as ‘interference’. During the expression stage, crRNAs are produced after processing the primary transcript (precursor CRISPR RNA or *pre-crRNA*) generated from the CRISPR locus (Mojica et al. 1993; Brouns et al. 2008). Like the eukaryotic interference RNA (RNAi) system, CRISPR-Cas utilises small guide RNA (crRNA) molecules to recognise complementary sequences (Brouns et al. 2008). However, in addition to RNA (Abudayyeh et al. 2016), CRISPR-Cas binds and cleaves target DNA sequences (Marraffini and Sontheimer 2008; Garneau et al. 2010) during the interference stage.

Apart from the crRNA guides and the Cas proteins that participate in all stages of the CRISPR-Cas mechanism, other main components are needed for CRISPR-based immunity: the leader sequence, the protospacer adjacent motif (PAM) and, in some CRISPR-Cas variants, the trans-activating CRISPR RNA (tracrRNA) or the short-complementarity untranslated RNAs (scoutRNAs). The *leader* is a sequence conserved at one end of CRISPR arrays that co-evolves with repeat sequences (Bult et al. 1996). The main promoter of the CRISPR locus (Brouns et al. 2008; Pul et al. 2010; Pougach et al. 2010) and motifs related to recognition of the spacer integration site (Rollie et al. 2015; Wei et al. 2015; Nuñez et al. 2016; Yoganand et al. 2017; McGinn and Marraffini 2019) are in the leader. *PAM*s are short sequences (typically 2 to 5 nt) at the end of the protospacers (Bolotin et al. 2005) of many CRISPR-Cas systems (Mojica et al. 2009; Shah et al. 2013). PAMs are necessary for the efficient recognition of protospacers by Cas proteins during the adaptation and interference stages (Gleditzsch et al. 2019). The requirement for PAMs prevents self-targeting of the CRISPR array as alternative sequences are present in the corresponding location next to the spacers (Weissman et al. 2020). Both *tracrRNA* and *scoutRNA* are small RNAs encoded in some CRISPR-Cas types, which partially hybridise with the repeat in CRISPR RNAs, participating in crRNA maturation and target interference (Deltcheva et al. 2011; Jinek et al. 2012; Harrington et al. 2020).

Along with the spacer-matching sequences found in transmissible genetic elements, protospacers were initially located in non-mobile chromosomal regions, suggesting that CRISPR might be playing in-house roles (Mojica et al. 2005). Indeed, as is the case of the immune system in mammals (Sattler 2017), non-canonical functions have been proven for the prokaryotic adaptive system since the initial demonstration of its protective action (Wimmer and Beisel 2020).

The biochemical characterisation of a few CRISPR-Cas systems in the late 2000s and early 2010s enabled easily programmable DNA targeting (Gasiunas et al. 2012; Jinek et al. 2012), providing tools for genome editing, notably those based on Cas9 (Cong et al. 2013; Mali et al. 2013), and for the regulation of gene expression (Bikard et al. 2013). They also allowed the implementation of sequence-specific anti-microbials (Bikard et al. 2014). More recently, mainly thanks to the discovery of novel CRISPR-Cas systems, the spectrum of CRISPR-based devices and applications extended to RNA targeting, molecular diagnostics, epigenetic modification or guided transposition, among others (East-Seletsky et al. 2016; Mojica and Montoliu 2016; Gootenberg et al., 2017; Chavez and Qi 2019; Liu et al. 2020a; Sun et al. 2020b).

Reviews covering CRISPR-Cas have been numerous over the last years in an attempt to capture the growing diversity of CRISPR/Cas configurations and the newly discovered functions and mechanistic peculiarities. At present, CRISPR is a very productive and fast-moving field of research whose updates are followed closely by the large CRISPR community devoted to understanding its biology and, beyond basic researchers, by those interested in applications of CRISPR-based technology. In this manuscript, we summarise the fundamentals of native CRISPR-Cas systems and further elaborate on lesser-known biological aspects, such as the complexities of their regulation and the diverse non-canonical functions they play.

## CRISPR-Cas diversity and classification

Although initial identification of CRISPR-Cas components pointed at a limited diversity of these systems and a general mechanism of action (Mojica et al. 2000; Jansen et al. 2002; Haft et al. 2005; Makarova et al. 2006; Barrangou et al. 2007), in-depth analyses of the increasing genomic and metagenomic data have demonstrated a staggering variety in CRISPR-Cas systems (Dwarakanath et al. 2015; Al-Shayeb et al. 2020; Pinilla-Redondo et al. 2020). Early classification schemes, relying mainly on just comparing a subset of *cas* genes (notably *cas1*), were gaining in complexity (Haft et al. 2005; Makarova et al. 2006). Cataloguing efforts of CRISPR-Cas elements focused on establishing robust criteria to reflect the phylogeny of the different systems. Thus, Makarova and co-workers recommended in 2011 a polythetic classification strongly supported by evolutionary relationships between CRISPR-Cas components (Makarova et al. 2011). Later, a critical layer was added to the categorisation efforts after defining the functional modular organisation of the CRISPR-*cas* locus (Makarova et al. 2015). According to the activities carried out by the Cas proteins, four modules of CRISPR-associated genes (sometimes with shared members) were differentiated: (i) adaptation, (ii) expression, (iii) interference or effector and (iv) signal transduction or ancillary (a combination of different accessory genes with unknown or tentatively assigned functions) modules. Subsequently, the comparison among the effector modules became the main classification principle considering Cas sequence similarity, *cas* locus architecture, the phylogeny of conserved Cas proteins, characteristics of other CRISPR associated elements and, ultimately, available experimental data. This complexity of criteria has resulted in a dynamic classification that must be regularly amended as new data are provided (Lange et al. 2013; Burstein et al. 2017; Harrington et al. 2017; Shmakov et al. 2017a; Yan et al. 2018, 2019; Makarova et al. 2018; Pausch et al. 2020).

**Fig. 1:**
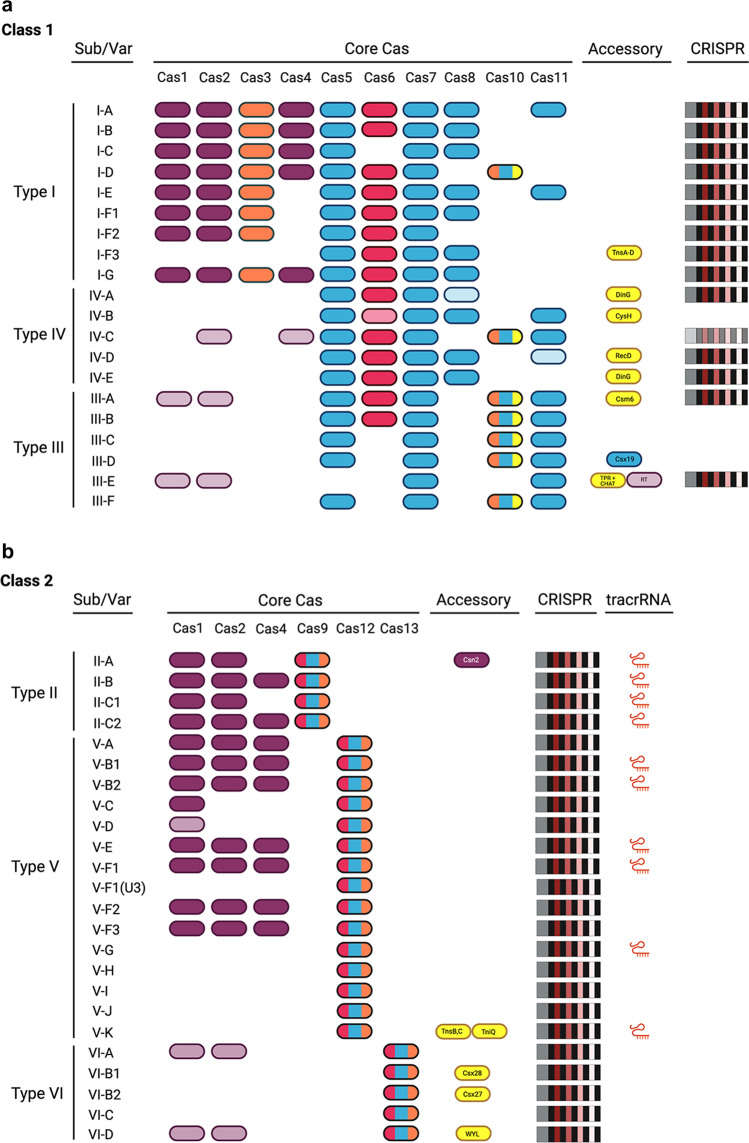
Components of CRISPR-Cas systems. The presence of CRISPR array (CRISPR), trans-activating crRNA (tracrRNA), and genes encoding either core Cas proteins (Core Cas) or accessory proteins/domains (Accessory) involved in ancillary functions (yellow) are shown for class 1 (**a**) or class 2 (**b**) CRISPR-Cas subtypes and variants (Sub/Var). Core proteins are coloured based on their role in adaptation (burgundy), crRNA biogenesis (red), target binding (blue) and target cleavage (orange), according to Makarova et al. (2020), Pausch et al. (2020) and Pinilla-Redondo et al. (2020). Constituents that are not invariably present are represented with fainter colours. Genes encoding proteins that contribute multiple functions are depicted with colour schemes consistent with the colour code assigned to each activity. TPR, tetratricopeptide repeat; CHAT, protease domain of the caspase family; RT, reverse transcriptase domain; TnsA-D and TniQ, transposition-related proteins; WYL, protein with the WYL domain

The current classification of the CRISPR-Cas systems (Pinilla-Redondo et al. 2020; Makarova et al. 2020; Pausch et al. 2020) comprises two classes (class 1 and class 2), six types (marked with Roman numbers, from Type I to Type VI) and over 30 subtypes (denoted by letters: I-A to I-F, IV-A to IV-E, III-A to III-F, II-A to II-C, V-A to V-K and VI-A to VI-D; provisionally classified systems are labelled with U), some also including multiple recognised variants (indicated with Arabic numerals). Figure 1 shows the typical components of the classified CRISPR-Cas systems.

The interference or effector module, responsible for target recognition, encodes for either a multiprotein effector complex (class 1 systems: types I, III and IV; termed Cascade in type I systems and Cmr/Csm in type III, the type IV complex does not have a specific name) or a single effector protein (class 2: types II, V and VI).

The class 1 effector complexes invariably comprise a Cas5 subunit and multiple subunits of Cas7, in addition to a small subunit (collectively denoted Cas11) and a large subunit (Cas8 or Cas10 in type I and III systems, respectively) (Jackson et al. 2014; Osawa et al. 2015). Some type I systems also have a Cas6 homolog associated with the complex (Haurwitz et al. 2010; Sashital et al. 2011). In type I systems, the crRNA-effector complex (surveillance complex) recruits an effector nuclease (Cas3) for target cleavage (Brouns et al. 2008). In other systems (type III and class 2), the effector proteins/complexes themselves are responsible for target cleavage (Jinek et al. 2012; Shmakov et al. 2015; Yan et al. 2019). Cas proteins involved in the interference stage seem to be absent in most type IV systems (Pinilla-Redondo et al. 2020).

CRISPR-Cas types of class 2 are distinguished by the single-protein effector associated with the system, namely, Cas9, Cas12 or Cas13 for type II, type V and type VI, respectively. The three protein families differ in the number, type and architecture of the nuclease(s) domain(s): type II and type VI effector proteins contain two nuclease domains (HNH and RuvC in the case of Cas9, two HEPN domains in Cas13), whereas those of type V have just one (RuvC) (Shmakov et al. 2015; Abudayyeh et al. 2016).

The adaptation module comprises the genes encoding enzymes involved in spacer acquisition, including Cas1 (fused to a reverse transcriptase domain in some type III and type VI systems), Cas2 (Cas1 and Cas2 are present in all the adaptation-proficient, autonomous systems), Cas4 (found in many type I, II and V systems) and Csn2 (exclusive to subtype II-A systems). Nevertheless, some system subtypes, notably within type IV and at a lower proportion in type III and VI systems, are devoid of any genes of the adaptation module (see section below on ‘Adaptation’).

The expression module deals with cleavage of the pre-crRNA and processing into mature crRNAs. Whereas this role is played by a dedicated Cas protein associated with many class 1 systems (see section below on ‘crRNA biogenesis’), class 2 involves a catalytic domain of the effector protein and, at least in type II systems, non-Cas ribonucleases.

Orphan CRISPR arrays and a range of unclassifiable, intermediate and minimal CRISPR-Cas configurations exist (Shmakov et al. 2020a; Pourcel 2020), suggesting degeneration of the CRISPR-Cas systems (Hermans et al. 1991; García-Gutiérrez et al. 2015; Chen et al. 2019). There is evidence that at least some of these apparently incomplete systems are functional (see below).

## The general CRISPR-CAS mechanism

**Fig. 2:**
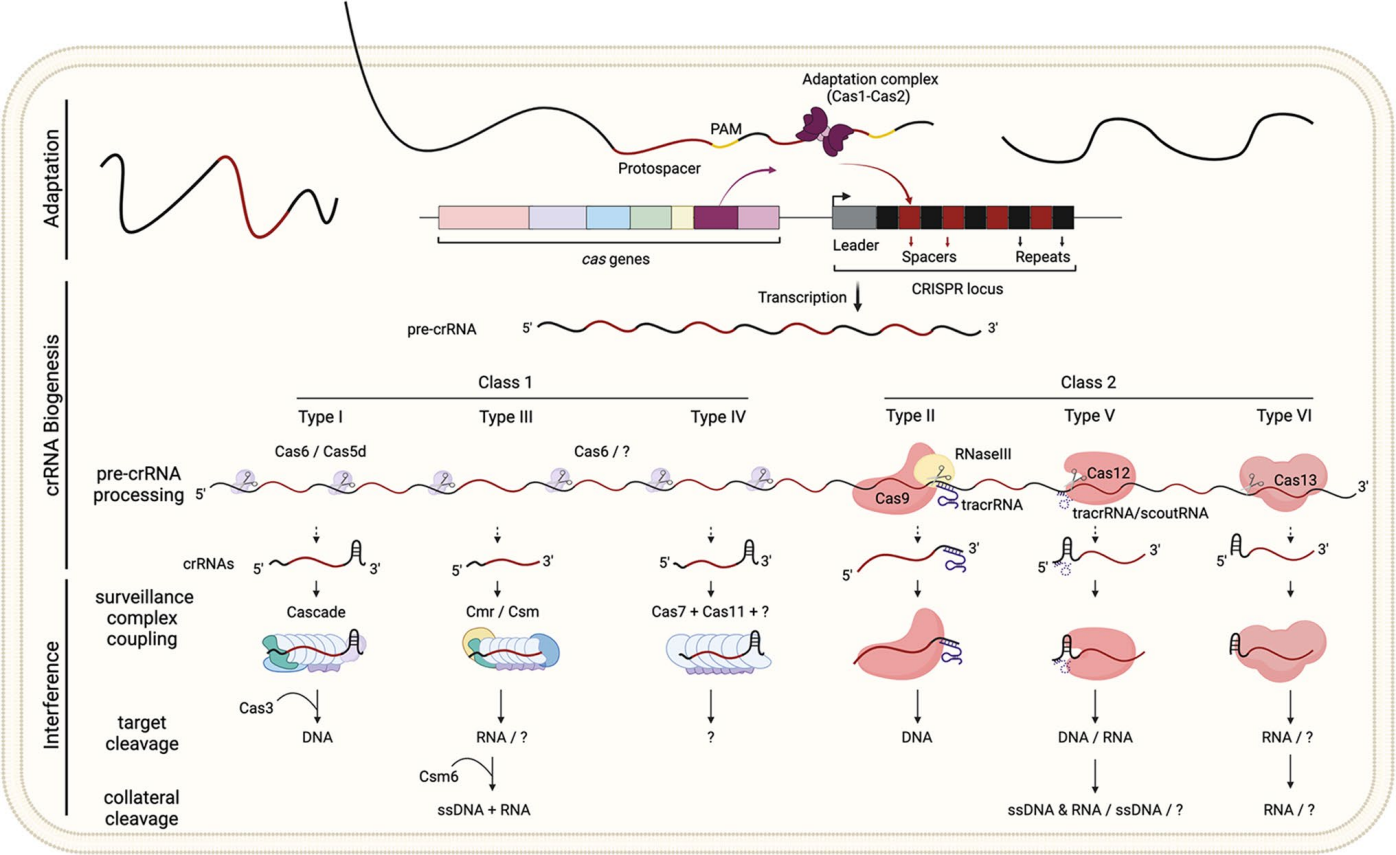
Schematic representation of the Adaptation, crRNA biogenesis and Interference stages of the canonical CRISPR-Cas mechanism. Sequences, typically derived from protospacers located next to a protospacer adjacent motif (PAM), are captured and processed by the adaptation complex (composed of at least Cas1 and Cas2 subunits). Non-Cas proteins may assist pre-spacer processing (not shown). The processed fragments are then preferentially integrated as spacers at the leader-repeat junction of the CRISPR loci by the adaptation complex (Adaptation stage). crRNAs are generated during the crRNA biogenesis stage after cleavage of the CRISPR array’s transcript (pre-crRNA). This cleavage is catalysed by different proteins depending on the system type. In some cases, subsequent maturation of the crRNAs is performed by either Cas or non-Cas exonuclease activities (see text for details). For pre-crRNA and target cleavage, type II systems and some type V subtypes require other CRISPR RNAs (tracrRNA or scoutRNA) that remain hybridised to the partially complementary crRNA. tracrRNA and scoutRNA are drawn with dashed lines when they are involved in only some subtypes of a given CRISPR-Cas type. During the interference stage, mature crRNAs bound to the effector complex (class 1) or protein (class 2), base pair with sequences complementary to the spacer region in RNA (type III, type VI and some type V systems) or DNA (the remaining types) target molecules. The recruitment of endonucleases (i.e. Cas3 in type I) or the activation of nuclease domains in the surveillance complex (other types) after target binding will trigger specific target cleavage. In types III, V and VI, target cleavage or binding leads to collateral unspecific cleavage of RNA (type VI systems), ssDNA (some type V systems) or both ssDNA and RNA (type III systems). Surveillance complex components and substrate identity have not been established for some system types or subtypes (indicated with a question mark)

Three stages have been identified in the generation of CRISPR-Cas immunity, namely (i) adaptation, (ii) crRNA biogenesis and (iii) interference (Fig. 2). This mechanism involves CRISPR RNAs and core Cas proteins encoded by various gene modules (as discussed above and recently reviewed by Nussenzweig and Marraffini 2020). In addition, the functionality of systems devoid of some of these components, both working autonomously and relying on either auxiliary proteins or CRISPR/Cas activities provided by systems that co-occur in the cell, has also been documented. Most of these atypical systems follow the adaptive, RNA-guided, nucleic-acid targeting and cleavage scheme, thus being considered programmable nuclease systems. Nevertheless, others result in alternative outcomes, such as guided transposition or targeting without cleavage. These alternatives to the canonical process will be covered later in this manuscript.

### Adaptation

Adaptation is the first step of the CRISPR-Cas mechanism, where a molecular memory is generated (for a recent review, see Mosterd et al. 2021). During this process, prespacers derived from protospacer-containing sequences are integrated into the CRISPR array as new spacers. Two models of CRISPR adaptation have been described, called primed and naïve (Datsenko et al. 2012; Yosef et al. 2012). Cas proteins of both the adaptation and effector module participate in primed adaptation, leading to a biased acquisition of spacers derived from the genetic element carrying the targets of pre-existing spacers. In contrast, naïve adaptation only requires adaptation machinery, and the selection of protospacers is independent of previous acquisitions. Both naïve and primed adaptations have been experimentally confirmed for the subtype II-A CRISPR-Cas system from *Streptococcus mutans* and diverse type I systems of *Escherichia coli* (subtype I-E), *Legionella pneumophila*, *Pseudomonas aeruginosa* and *Geobacter sulfurreducens* (Datsenko et al. 2012; Díez-Villaseñor et al. 2013; Savitskaya et al. 2013; Semenova et al. 2016; Rao et al. 2017; Almendros et al. 2019). In addition, primed but not naïve adaptation was detected in subtype I-B of *Haloarcula hispanica* (Li et al. 2014a, b) and the subtype I-F of *Pectobacterium atrosepticum* and *E. coli* (Richter et al. 2014; Vorontsova et al. 2015). On the contrary, naïve but not primed adaptation has been reported for subtype I-A from *Sulfolobus solfataricus* and *Sulfolobus islandicus*, II-A of *Streptococcus agalactiae* and III-B in *S. solfataricus* (Deveau et al. 2008; Erdmann et al. 2014; Heler et al. 2015; Shiimori et al. 2017; Nussenzweig et al. 2019; Artamonova et al. 2020).

Cas1 and Cas2 are the essential Cas proteins for adaptation (Makarova et al. 2015; Koonin et al. 2017). The minimal adaptation complex of type I systems comprises two Cas1-dimers joined by a Cas2-dimer (Nuñez et al. 2014, 2015). Nuclease and integrase activities involved in the spacer integration process are provided by Cas1 (Wiedenheft et al. 2009; Babu et al. 2011), while Cas2 has a structural function (Wang et al. 2015). In some CRISPR-Cas systems, additional Cas proteins and activities participate in adaptation: Cas4 in some type I, II and V subtypes (Heler et al. 2015; Hudaiberdiev et al. 2017; Rollie et al. 2018; Kieper et al. 2018; Shiimori et al. 2018; Lee et al. 2019; Almendros et al. 2019), Cas9 in type II, together with Csn2 in subtype II-A systems (Wei et al. 2015) and the reverse transcriptase domains fused to Cas1 (RT-Cas1) in type III and VI variants (Silas et al. 2016; Toro et al. 2019; González-Delgado et al. 2019).

Before their integration, spacer precursors must be recruited by the adaptation complex. Most CRISPR-Cas systems select protospacers after recognising the PAM. Specific motifs located in the PAM region next to the interference target (therefore also referred to as PAM) are likewise required for efficient target recognition and cleavage performed by the effector Cas proteins (Deveau et al. 2008; Datsenko et al. 2012; Swarts et al. 2012; Shah et al. 2013). Although the adaptation and the interference consensus PAMs may differ for a given CRISPR-Cas system (Almendros et al. 2012; Shah et al. 2013), there is a strong preference for acquiring spacers from sequences flanked by a PAM that is compatible with the interference machinery (Yosef et al. 2012, 2013; Díez-Villaseñor et al. 2013; Nuñez et al. 2014; Levy et al. 2015). Bona fide PAMs have not been identified next to protospacers of most type III systems, which is not surprising since type III systems usually display PAM-independent interference. Remarkably, in those type III systems associated with RT-Cas1, ssRNA transcripts are captured and converted into DNA once incorporated into the CRISPR array (Silas et al. 2016; González-Delgado et al. 2019).

In *E. coli*, although induction of Cas1 and Cas2 of its I-E system triggers naïve acquisition of spacers mainly derived from resident plasmids, chromosomal DNA sequences are also captured (Levy et al. 2015). These chromosomal spacers preferentially derive from the origin of replication and, notably, the *terminus* region. In this regard, it has been proposed that ssDNA fragments generated upon repair of DNA double-strand breaks, like those produced during replication (Smith 2012), are the primary source of prespacers (Levy et al. 2015; Radovčić et al. 2018). This replication-related origin of naïve spacers would partially explain the prevailing acquisition of sequences from plasmids versus the less often replicated chromosomes. However, it is unknown whether the Cas1-Cas2 complex captures ssDNA or dsDNA molecules, and it remains to be elucidated when and how the dsDNA spacers are generated from repair-derived ssDNA fragments. Concerning primed adaptation, it has been shown that the nuclease activity of Cas3, probably associated with the adaptation machinery in the so-called primed adaptation complex, produces prespacers in I-E and I-F systems (Shiriaeva et al. 2020; Musharova et al. 2021).

Once loaded into the Cas1-Cas2 adaptation complex, the prespacer ends must be trimmed to the size of the spacer. In subtypes I-A and I-C, pruning of the prespacers’ 3′ ends is carried out by the Cas4 protein (Rollie et al. 2018; Lee et al. 2019). However, many CRISPR-Cas systems lack Cas4. Subtype I-E in *S. thermophilus* is one of the few systems where Cas2 is fused to a DnaQ domain. In vitro studies have shown that this domain has exonuclease activity and that the I-E adaptation complex of this species can process and integrate duplex oligonucleotides with 3′ protruding ends (Drabavicius et al. 2018). In the case of the I-E system of *E. coli* (devoid of Cas4 and with a Cas2 protein that does not exhibit exonuclease activity), DnaQ and ExoT exonucleases, as well as the proofreading subunit of the DNA polymerase III, have been suggested to be involved in the prespacer trimming (Kim et al. 2020; Ramachandran et al. 2020). According to the model proposed, once the prespacer is loaded into the Cas1-Cas2 complex and the PAM is recognised, host nucleases will degrade the ends of the prespacer until reaching the region protected by the complex (Yoganand et al. 2019).

dsDNA prespacers are preferentially integrated into the CRISPR locus at the leader proximal end of the CRISPR array (Barrangou et al. 2007). The adaptation machinery of type II systems spots this integration site after recognising the leader-anchoring sequence (LAS) within the leader (Wei et al. 2015). Similarly, in subtype I-E, specific sequences in the leader and the repeat are bound by the integration host factor (IHF) protein, which generates a docking site for the Cas1-Cas2 complex upon bending this DNA region at the leader-repeat junction (Nuñez et al. 2016; Yoganand et al. 2017). Once in place, the Cas1-Cas2 complex catalyses direct nucleophilic attack of the 3′-OH ends of the prespacer at the leader-repeat junction and, subsequently, at the repeat-spacer boundary, resulting in a dsDNA spacer flanked by single-stranded repeats that must be repaired and ligated. Neither the ligase nor the DNA polymerase involved in this process has been identified so far. However, *E. coli* DNA polymerase I mutants cannot acquire spacers, uncovering this protein as a putative candidate (Ivančić-Bace et al. 2015). Meanwhile, a recent study suggested that primase-polymerase homologues associated with some III-A and III-B CRISPR-*cas* loci might participate in spacer adaptation (Zabrady et al. 2021).

### crRNA biogenesis

The biogenesis of crRNAs is a crucial step for target recognition and cleavage. CRISPR arrays are usually transcribed from a single promoter located in the leader, generating the pre-crRNA. Later, this transcript is cleaved into small RNAs. Finally, at least in some systems, these RNA molecules are trimmed to create the mature crRNAs that will become a functional component of the surveillance complex. Occasionally, transcription is observed from promoters within the CRISPR array, either in spacers or in repeats (Lillestøl et al. 2009; Wurtzel et al. 2010; Deng et al. 2012; Zhang et al. 2013).

Class I crRNA maturation is performed by Cas6 protein except for subtypes I-C, III-C and III-D (Carte et al. 2008; Haurwitz et al. 2010; Gesner et al. 2011; Sashital et al. 2011; Nam et al. 2012; Richter et al. 2012; Garside et al. 2012; Özcan et al. 2019). The partially palindromic sequences of the repeats that constitute the CRISPR arrays in types I-D, I-E and I-F adopt a stem-loop structure in the transcribed pre-crRNA. Cas6 recognises this hairpin structure and cleaves the pre-crRNA downstream the loop (Haurwitz et al. 2010; Gesner et al. 2011; Sashital et al. 2011; Nam et al. 2012). The resulting crRNA comprises a spacer flanked by a short repeat-derived sequence at the 5′ end and the stem-loop at the 3′ end. Cas6 remains bond to the crRNA loop and serves as a scaffold for the multimeric effector complex (Jore et al. 2011; Sashital et al. 2011). For subtype I-C, the crRNA maturation occurs similarly, but the function of Cas6 is performed by Cas5d (Nam et al. 2012; Garside et al. 2012). In subtypes I-A and I-B, CRISPR repeats are not palindromic, and in III-A and III-B systems, hairpins are unstable (Koonin et al. 2017). In these cases, dimers of Cas6 generate a conformational change in the pre-crRNA, creating a hairpin-like secondary structure and cleaving within the repeat (Richter et al. 2013; Shao and Li 2013; Reeks et al. 2013; Sefcikova et al. 2017). Later, Cas6 is released from the crRNAs whose 3′ ends are trimmed by a protein not yet identified (Carte et al. 2008, 2010; Hatoum-Aslan et al. 2011). The sequence of type III Cas6 proteins resembles that of types I-A and I-B, and, accordingly, their pre-crRNAs are processed following the same pattern. For subtypes III-C and III-D, as well as III-F, where no homologs to *cas6* have been described, Cas5 orthologs could be responsible for pre-crRNA cleavage (Behler and Hess 2020). The maturation of crRNA in type IV has not been studied in detail. Still, for subtype IV-A, it was established that the Cas6 homolog Csf5 protein is responsible for pre-crRNA maturation (Özcan et al. 2019).

Cleavage of pre-crRNA and maturation of crRNA in class II systems relies on the effector protein and, depending on the CRISPR-Cas type, on other genetic elements or proteins. Type II systems require tracrRNA (Deltcheva et al. 2011; Gasiunas et al. 2012; Zhang et al. 2013; Shmakov et al. 2015). Once the tracrRNA anneals with its complementary sequence in the crRNA repeats region, in subtypes II-A and II-B, Cas9 binds and stabilises the crRNA:tracrRNA structure. Later, the homing ribonuclease RNaseIII cleaves the crRNA within the repeat at the 3′ end, and an unknown nuclease processes the 5′ end (Deltcheva et al. 2011). Instead, a tracr-dependent but RNaseIII-independent mechanism was discovered in II-C systems from *Campylobacter jejuni*, *Neisseria meningitidis* and *Neisseria lactamica* (Dugar et al. 2013; Zhang et al. 2013)*.* In these cases, crRNAs are produced individually due to the presence of promoters within the repeats that generate short RNA molecules. In type V, the effector protein Cas12 is responsible for pre-crRNA cleavage. Subtype V-A uses a tracr-independent mechanism to process the pre-crRNA where Cas12, upon recognition of the hairpins formed in the repeat regions, cleaves within them to generate the crRNA. In the other type V subtypes, either tracrRNA or scoutRNAs are needed to efficiently process the crRNA (Yang et al. 2016; Liu et al. 2019a; Harrington et al. 2020). Meanwhile, the mechanism of pre-crRNA cleavage in subtype V-F remains unknown (Behler and Hess 2020). Cas13 RNA-effector protein of type VI can also cut within the pre-crRNA without the tracrRNA, relying on the recognition of the secondary structure of the repeat region in a similar way to type I, cleaving upstream of the hairpin (Shmakov et al. 2015; Abudayyeh et al. 2016; East-Seletsky et al. 2017).

In addition to RNase III, other host proteins may participate in crRNA maturation. In the type III-B of *Synechocystis* sp. PPC 6803, RNase E is recruited for pre-crRNA processing and cleaves it within the repeat (Behler et al. 2018). Polynucleotide phosphorylase (PNPase) cleaves the pre-crRNA in collaboration with Cas6 in the III-A system of *S. epidermidis* (Samai et al. 2015; Chou-Zheng and Hatoum-Aslan 2019), and in the I-B system of *Haloferax volcanii*, RNase Z and RNase P were repurposed to successfully cleave pre-crRNA in a modified strain lacking Cas6 (Maier et al. 2015).

Further investigations will be needed to decipher which yet unknown proteins are involved in the trimming of the crRNA and how the crRNA maturation is achieved in systems lacking Cas6 homologs, RNase III or tracrRNA/scoutRNA.

### Interference

Interference is the last stage of the CRISPR-Cas mechanism, where the Cas effector proteins form a surveillance complex with CRISPR RNAs that guide the proteins to a target sequence complementary to the crRNA spacer region (Hille and Charpentier 2016). Firstly, in the case of PAM-dependent dsDNA targeting systems, the surveillance complex scans DNA molecules in search of PAMs. Once a cognate motif is identified, dsDNA is locally unwound. Then, the PAM-proximal positions in the so-called seed sequence (Semenova et al. 2011; Swarts et al. 2017) are probed for complementarity with the crRNA spacer and, subsequently, interrogation of base-pairing proceeds beyond that region. Then, as the RNA:DNA hybrid forms, the non-complementary DNA strand is displaced, forming an R-loop structure (Stella et al. 2017; Xiao et al. 2017). Finally, if sufficient hybridisation is reached, the target becomes fully accessible to the nuclease effector, licensing cleavage.

Even though this can be considered the typical mechanism of interference against dsDNA targets, there are prominent differences among CRISPR-Cas subtypes related to PAM requirement and location, as well as to the nucleic acids that are targeted (dsDNA, ssDNA and/or RNA sequences) and cleaved (only the target or, in addition, unspecific RNA and/or DNA sequences).

For a comprehensive review on class 1 effectors, see Liu and Doudna (2020). Among type I systems, interference has been studied in greater detail for subtype I-E of *E. coli*. First, the Cascade surveillance complex recognises a downstream (taking the target strand as a reference) PAM in duplex form (Hayes et al. 2016). After R-loop formation, the Cas3 protein is recruited by the complex (Xiao et al. 2017). Then Cas3 nicks within the displaced ssDNA at the R-loop and catalyses subsequent cuts as it translocates along this strand (He et al. 2020). In contrast to type I, the surveillance complex of at least some type III subtypes (III-A, B, C) degrades ssDNA and ssRNA. Through the RNase activity of the Cas7-like subunits in the Cmr/Csm complex, crRNA-complementary sequences in RNA molecules are degraded. The binding of the complex to the RNA target activates the ssDNA nuclease activity of Cas10 (another integral part of the complex). It was revealed in some III subtypes that cyclic oligoadenylates (cOAs) produced by Cas10 activate a separate Csm6 nuclease effector to degrade non-specific RNAs (Kazlauskiene et al. 2017; Niewoehner et al. 2017; Jia et al. 2019). Most type III systems do not require specific motifs flanking the target for efficient recognition, and, accordingly, clear evidence of functional seed sequences has not been found (Marraffini and Sontheimer 2010; Osawa et al. 2015; Estrella et al. 2016). However, the type III-B system from *Pyrococcus furiosus* recognises a protospacer flanking sequence or PFS (defined as protospacer flanking site by some authors) next to the RNA target (Foster et al. 2020). Interestingly, hybridisation between PFS and the crRNA prevents DNase and cOA production activities but still licences specific RNA degradation. Regarding type IV systems, the first experimental proof of interference in vivo was recently reported by Crowley and co-workers (Crowley et al. 2019). However, the identity of the target (DNA or RNA) and the mechanism involved remain undisclosed (Pinilla-Redondo et al. 2020).

In class 2 systems, type II and some type V subtypes require a hybrid RNA guide composed of tracrRNA and crRNA molecules (Jinek et al. 2012; Shmakov et al. 2015; Liu et al. 2019a; Yan et al. 2019). Subtype V-C and V-D systems, together with crRNA, require scoutRNAs for target cleavage (Harrington et al. 2020). While Cas9 recognises PAMs located downstream of the non-target strand (Gasiunas et al. 2020), PAMs located at the opposite flank are identified on both strands of the DNA target by Cas12 (Shmakov et al. 2015). Type II and some type V systems target dsDNA and cleave the two strands. Other type V subtypes target ssDNA, both dsDNA and ssDNA, or ssRNA (Harrington et al. 2018; Yan et al. 2019; Karvelis et al. 2020; Pausch et al. 2020). Remarkably, after specific target cleavage, at least some Cas12 nucleases develop collateral ssDNA or ssDNA and RNA nuclease activity (Yan et al. 2019). Meanwhile, Cas13 effector proteins of type VI systems degrade non-specific RNAs upon identification of the target RNA (Abudayyeh et al. 2016; Smargon et al. 2017; Liu et al. 2017; Yan et al. 2018). Although no canonical PAM sequence is required for efficient interference by these systems, some Cas13 variants recognise a PFS region downstream of the target (Leenay and Beisel 2017).

## Functions of CRISPR-Cas systems

The most apparent benefit for a cell from encoding adaptive immunity machinery such as the one provided by the CRISPR-Cas systems is protection against viruses: the genome of invading viruses and resident proviruses entering a lytic cycle can be specifically degraded, preventing cell damage and the eventual spread of virions. Furthermore, the genetic memory licenced by integrating new spacers derived from the infecting virus will further protect the descendants of the adapted cell for generations, thus perpetuating the anti-virus outcome. Similarly, looking at plasmids as parasitic agents that may place a burden on the cell, the immunity concept also covers interference against these transmissible molecules. Moreover, type IV systems primarily found in plasmids preferentially target sequences in other plasmids, suggesting that this CRISPR-Cas type is specialised in competition between these kinds of molecules (Pinilla-Redondo et al. 2020).

Immunity at the cell and population level is considered the primary purpose of CRISPR-Cas (Edgar and Qimron 2010; Cady et al. 2012; Strotskaya et al. 2017; Watson et al. 2019; Deem 2020). In fact, since 2007 (Barrangou et al. 2007), many studies have proven this defensive role in prokaryotes. Thus, both virus resistance and plasmid cleavage has been used as a recurrent strategy to assess CRISPR-Cas activity (Marraffini and Sontheimer 2008; Garneau et al. 2010; Westra et al. 2013b; Almendros and Mojica 2015; Crowley et al. 2019; Wheatley and MacLean 2020).

Otherwise, invasive mobile genetic elements (iMGEs) such as viruses and plasmids represent an opportunity to acquire foreign DNA. Hence, immunity against these elements has the potential to restrict horizontal gene transfer (HGT). Indeed, it has been shown that CRISPR-Cas systems constitute a barrier to HGT in diverse bacteria and archaea, preventing conjugation, transduction and natural transformation and thereby influencing traits such as bacterial virulence and drug resistance or even microbial speciation (Marraffini and Sontheimer 2008; Mojica and Díez-Villaseñor 2013; Turgeman-Grott et al. 2019; Zhou et al. 2020; Kamruzzaman and Iredell 2020; Wheatley and MacLean, 2020). Interestingly, the inverse has also been reported: recombination between CRISPR spacers in bacterial genomes and their targets in invading bacteriophages facilitates the transfer of CRISPR-Cas systems and adjacent regions through escape transduction particles, favouring, rather than dampening, HGT (Watson et al. 2018; Varble et al. 2019).

However, works assessing the relevance of CRISPR-Cas in the battle against viruses and lateral gene dissemination are scarce. Hence, the actual impact of these systems in natural environments remains to be firmly established (Westra and Levin 2020; Martínez Arbas et al. 2021).

On the other hand, most spacers so far identified in prokaryotic genomes (i.e. chromosomes and resident plasmids) do not match known virus or plasmid sequences (Shmakov et al. 2017b, 2020b). In this context, functions other than the control of iMGEs have been reported for complete and partial CRISPR-Cas systems. These non-canonical activities range from regulatory tasks to guiding transposition events and result in virulence control or genome evolution, among many other outcomes. Some of these roles are just hypothetical, even though well substantiated. For instance, the cOAs synthesised by type III Cas10 (Kazlauskiene et al. 2017) might act as extracellular messengers that enable bacterial communication, integrating Cas proteins within cell signalling pathways. Other non-canonical functions for CRISPR-Cas encoded in prokaryotic genomes have been identified and will be discussed below. Given the peculiarities of the roles played by CRISPR and Cas found in prokaryotic viruses, their functions will be addressed in a dedicated section.

### Cytotoxicity, cell dormancy and regulation of gene expression

The indiscriminate degradation of nucleic acids exhibited by some CRISPR-Cas systems after recognising the specific target may result in cell suicide (Hale et al. 2009, 2012; Abudayyeh et al. 2016; Liu et al. 2017).

Furthermore, the observation that many spacers perfectly match fragments within the carrier genome (i.e. self-targeting spacers) (Horvath et al. 2008, 2009; Stern et al. 2010) raised the possibility that CRISPR-Cas activity might have a variety of consequences in non-infected cells (reviewed in Wimmer and Beisel 2020). At present, full-matching self-targeting spacers and spacers with only partial complementarity to resident genomic sequences have been involved in downregulation and upregulation of expression affecting DNA repair, virulence, anti-microbial susceptibility or cell development processes (Newsom et al. 2021). However, in many cases, the underlying regulatory mechanism remains to be established (Table 1). For example, even though endogenous gene regulation unconnected to immunity was predicted as the primary function of the type II-B Cas2 protein in *Legionella pneumophila* (Gunderson and Cianciotto 2013), and an orphan CRISPR locus in *Listeria monocytogenes* (Mandin et al. 2007), further studies are necessary to confirm this implication (Bozic et al. 2019).

**Table 1:** Cas proteins involved in regulation of gene expression through an unestablished mechanism

Host	Subtype^a^	Cas^b^	Main processes affected	Reference
*Sulfolobus islandicus*	I-A	Csa3a	DNA repair CRISPR adaptation	Liu et al. (2017)
*Streptococcus mutans*	I-C	Cas3	Virulence Antimicrobial resistance	Tang et al. (2019)
*Porphyromonas gingivalis*	I-C	Cas3	Virulence	Solbiati et al. (2020)
*Myxococcus xanthus*	I-C	Cas8c Cas7 Cas5	Cell development	Rajagopalan and Kroos (2017)
*Salmonella enterica*	I-E	Cas3	Virulence	Cui et al. (2020)
Group B *Streptococcus*	II-A	Cas9	Virulence	Spencer et al. (2019)
*Streptococcus pyogenes*	II-A	Cas9	Virulence	Gao et al. (2019)
*Streptococcus mutans*	II-A	Csn2	Virulence	Zhang et al. (2020)
*Riemerella anatipestifer*	II-C	Cas9	Virulence	Wang et al. (2019)
*Campylobacter jejuni*	II-C	Cas9	Virulence	Shabbir et al. (2018)
*Neisseria meningitidis*	II-C	Cas9	Virulence	Heidrich et al. (2019)
*Myxococcus xanthus*	III-B	RAMPs^c^	Cell development	Wallace et al. (2014)

^a^CRISPR-Cas subtype

^b^Cas proteins for which involvement in regulation has been experimentally demonstrated

^c^At least some proteins from among Cmr, Cas10 and Cas6

Better known illustrations of gene regulation executed by CRISPR-Cas systems through DNA or RNA targeting, and their consequences, are discussed below.

#### Regulation by DNA targeting

The first case of a non-canonical function played by CRISPR-Cas acting on DNA was reported for the type I-F system of *P. aeruginosa* (Zegans et al. 2009). The Cas3 nuclease, guided by a crRNA partially complementary to a resident prophage sequence, generated minor DNA damage instead of the processive degradation of the target characteristic of full-matching spacers (Xiao et al. 2018). Nevertheless, this DNA insult triggers the SOS response, which leads to de-repression of phage-related lysis genes (Cady and O’Toole 2011; Heussler et al. 2015). In this way, CRISPR-Cas activity indirectly induces the expression of proteins that can kill the cell, dampening dissemination of the carried phage and thus behaving as a population protective mechanism.

Through DNA targeting without cleavage, CRISPR-Cas can also achieve direct regulation of endogenous genes. This action is exemplified by Cas nucleases that bind DNA but are not able to cut it due to only partial complementarity with the crRNA, resulting in transcriptional silencing when the target is located near promoter regions (Ratner et al. 2019; Sampson et al. 2019). Indeed, an early work (Aklujkar and Lovley 2010) suggested that interaction of a crRNA from the I-E system of *Pelobacter carbinolicus* with a partially complementary sequence in the histidyl-tRNA synthetase gene (*hisS*) resulted in an attenuated histidyl-tRNA pool. Moreover, estimation of the amount of *hisS* transcript in a heterologous host carrying the targeting spacer hinted at a cleavage independent regulatory mechanism, ruling out the degradation of the *hisS* RNA and cleavage of the encoding DNA by the associated Cas nuclease. The type II CRISPR-Cas system in the pathogenic bacterium *Francisella novicida* typifies another well-substantiated case of gene expression regulation mediated by DNA-binding without cutting. It has been revealed that Cas9 transcriptionally represses endogenous genes through binding to the DNA targets, guided by a tracrRNA-scaRNA (small CRISPR/Cas-associated RNA) hybrid (Ratner et al. 2019; Sampson et al. 2019), rather than through RNA degradation as previously suggested (Sampson et al. 2013). This repression facilitates bacterial evasion of the innate immune system in infected mammalian hosts, enhancing virulence.

Cas proteins can also function as transcriptional activators upon binding to the gene promoters. For instance, the type I-A associated protein Csa3a from *S. islandicus* activates transcription of the adaptation *cas* genes and multiple repair genes such as those encoding DNA polymerase II, DNA polymerase IV, the NurA nuclease and the helicase HerA (Liu et al. 2017). Although the precise link between CRISPR and DNA repair has not been established, this case supports the synergy between CRISPR-Cas activity and the DNA repair process (see the ‘DNA repair’ section).

#### Repression through RNA cleavage

On the other hand, regulation of gene expression by some CRISPR-Cas systems is also achieved through RNA cleavage. In addition to the RNA-targeting CRISPR-Cas, some type II (Louwen et al. 2013; Sampson and Weiss 2013; Sampson et al. 2013; O’Connell et al. 2014; Dugar et al. 2018; Rousseau et al. 2018; Strutt et al. 2018) and type I (Li et al. 2016) systems that typically target DNA possess promiscuous nucleases able to bind and cut within RNA molecules, resulting in regulatory functions. In this context, it has been reported that *E. coli* subtype I-E Cascade binds ssRNA in vitro (Jore et al. 2011), and Cas3 can degrade ssRNA (Babu et al. 2011). However, evidence of such activities has not been provided in vivo. More recently, Li and co-workers showed that Cas3 protein and Cascade complex of *P. aeruginosa* PA14 (subtype I-F system) are involved in *lasR* mRNA degradation requiring just the presence of a PAM-like motif next to the *lasR* mRNA target and as little as 28% complementarity with the spacer (Li et al. 2016). Surprisingly, both sequence requisites are found in many other mRNAs encoded by the PA14 genome, raising questions such as whether this CRISPR system plays a significant role in regulating the abundance of individual mRNAs in the cell or, if *lasR* mRNA is the sole target (this question remains to be addressed), how specificity is achieved (Müller-Esparza and Randau 2017). In addition to type I, diverse subtype II-C and *S. agalactiae* II-A systems have also been reported to cleave RNA and, as in the case of the *P. aeruginosa* I-F system, efficient degradation of endogenous RNAs occurs despite only partial complementarity to naturally occurring spacers (Dugar et al. 2018; Ma et al. 2018; Rousseau et al. 2018; Strutt et al. 2018).

### Genome evolution

Genome evolution is the process of genetic variation in response to environmental variables. In prokaryotes, it occurs through point mutations, horizontal gene transfer and genome rearrangements. These events generate novel genotype variants which are transferred from a source cell to subsequent generations.

Prokaryotic evolution can be affected by CRISPR-Cas activity when it targets foreign genetic elements (e.g. dampening HGT) and when targets the host genome (self-targeting spacers), leading to autointerference. It has been estimated that self-targeting spacers account for 6% of the total pool identified in available sequenced genomes (Shmakov et al. 2017b). Thus, some incomplete, apparently non-functional CRISPR-Cas systems might have emerged because of self-targeting. Otherwise, mutations or rearrangements must occur in the target region to prevent autointerference and cell death, thus pushing genome evolution (Stern et al. 2010; Wimmer and Beisel 2020). This possibility has been experimentally demonstrated in a few cases. For example, induction of the type I-F CRISPR-Cas system in *P. atrosepticum* carrying self-targeting spacers resulted in deletions involving the target sequence (Vercoe et al. 2013). Likewise, in *S. thermophilus*, genome rearrangements of a chromosomal locus were also reported to happen at an increased frequency when the resident II-A CRISPR-Cas system targeted sequences located in that region (Selle et al. 2015; Cañez et al. 2019). Similar results have been reported for subtype I-B of *Haloferax volcanii* (Stachler and Marchfelder 2016) and subtypes I-A and III-B of *S. islandicus* (Li et al. 2015). Further proof of the concept that CRISPR-Cas immunity can contribute to bacterial diversity has been recently provided (Mo et al. 2021). The authors showed that the III-A CRISPR-Cas system in *Staphylococcus* species is mutagenic even in the absence of infecting agents. Moreover, increased host mutations occurred upon CRISPR-mediated targeting of lytic phages or plasmids when the associated Cas10 protein was active. These results suggested that the collateral ssDNA cleavage activity of Cas10 and the subsequent DNA repair would be responsible for random mutations in the chromosome, raising the possibility that other systems exhibiting non-specific ssDNase activity (i.e. Type V CRISPR-Cas) could modulate genome evolution in the same way.

Another remarkable case of CRISPR-driven evolution was proposed for the CRISPR-Cas I-E system from *P. carbinolicus* (Aklujkar and Lovley 2010), involving the previously mentioned (the ‘Cytotoxicity, cell dormancy and regulation of gene expression’ section) spacer matching a sequence within the *hisS* gene. CRISPR-mediated interference against the histidyl-tRNA-synthetase activity is expected to impair histidine-enriched proteins’ translation. Indeed, in contrast to closely related bacteria lacking such a *hisS*-interference capacity, *P. carbinolicus* cannot reduce Fe(III), a catalytic process that involves enzymes with high histidine content. Notably, transcription of *hisS* in a recombinant, closely related species carrying a *hisS*-targeting CRISPR-Cas system decreased compared to control strains without targeting capacity. Thus, CRISPR-mediated self-targeting might be responsible for the loss in this species of ancestral genes encoding proteins with high histidine content, having contributed significantly to its metabolic divergence from other members of the *Geobacteraceae* family.

CRISPR-Cas systems in prokaryotic cells might also accelerate the mutation rate of invasive genetic elements during infection. This effect has been demonstrated for a type II system in heterologous *E. coli* hosts exposed to a CRISPR-targeted bacteriophage T4 (Tao et al. 2018). Mutation frequencies in the phage genome were several orders of magnitude higher than the frequency observed in the absence of CRISPR-Cas activity. In this way, the CRISPR-Cas systems promote variability of the virus population providing selective advantages to infectious agents in parallel to the protective role played in the host.

### DNA repair

Mechanisms of genetic repair that rely on the arrangement of damaged DNA by gap-filling or ligation reactions link to the CRISPR-Cas mode of action.

For example, regarding the adaptation apparatus, the nuclease Cas1 of the I-E system from *E. coli* interacts with several repair system components (i.e. RecB, RecC, RuvB and RuvC), and it is actively involved in the cell rescue during DNA damage (Babu et al. 2011). Also related to spacer acquisition, it was reported that the II-A CRISPR-Cas associated protein Csn2 can inhibit DNA repair by the non-homologous end-joining (NHEJ) mechanism, explaining the low frequencies at which NHEJ repair and II-A CRISPR-Cas systems coexist within the same microbial genome (Bernheim et al. 2017). Because Csn2 binds the free DNA ends generated after cleavage produced by the adaptation complex at the spacer integration site (Nam et al. 2011; Arslan et al. 2013), it was proposed that repair inhibition is due to competition between Csn2 and the NHEJ-associated Ku protein for binding DNA ends to be repaired.

On another front, it has been shown that the type II effector protein Cas9 triggers the SOS-system response in the heterologous host *E. coli* as a collateral effect of its DNA-targeting (Cui and Bikard 2016). Interestingly, this SOS response leads to repairing the damaged genetic material through homologous recombination or, in the absence of donor DNA, large deletions due to the action of the RecBCD pathway.

These observations indicate a strong interconnection between CRISPR-Cas and DNA repair machinery, which merits further attention.

### Guided transposition

Transposons (Tn) are mobile DNA elements capable of excising and inserting themselves elsewhere in the genome through the activity of transposases assisted by other accessory proteins encoded by the element. The identity of the DNA targeted for integration by the TnsAB transposase complex of the prokaryotic Tn7 transposons is marked by either TnsD (targets the specific attachment site attTn7 in the chromosome) or TnsE protein (targets random sequences in the lagging DNA strand produced during plasmid replication) (Peters et al. 2017; Dimitriu et al. 2019).

Tn7-like transposons associated with I-B and I-F CRISPR-Cas systems were detected in silico in 2017 (Peters et al. 2017). Subsequently, association with similar transposons was also reported for subtype V-K systems (Strecker et al. 2019). These CRISPR-associated transposases (CASTs) consist of core transposase genes, either one (V-K and I-F systems) or two (I-B systems) *tniQ* genes (homologous to *tnsD*), a small CRISPR array and the genes encoding Cas effector proteins (Cascade proteins in type I systems or a nuclease-deficient Cas12 in type V systems), lacking adaptation module and effector nuclease (Cas3 in type I) activity (Peters et al. 2017; Faure et al. 2019a; Klompe et al. 2019; Strecker et al. 2019; Saito et al. 2021). A tracrRNA is also present in V-K CRISPR loci. Thus, the capacity to form surveillance complexes with guide RNA molecules is still maintained. Interestingly, TniQ proteins interact in the three subtypes with the surveillance complex to promote transposon integration next to sites targeted by the crRNA (Klompe et al. 2019; Strecker et al. 2019; van der Oost and Mougiakos 2020; Saito et al. 2021). RNA-guided transposition into specific sites of the host genome (homing transposition) involves crRNAs with either a truncated spacer encoded in a CRISPR array located away from the CRISPR-Cas locus (Subtype V-K; Saito et al. 2021) or spacers with low identity to the target, encoded by the cognate CRISPR array but flanked by diverged repeats (I-F systems; Petassi et al. 2020). Noteworthy, in addition to RNA-guided, homing transposition in I-B systems is elicited by a protein-target mechanism independent of crRNA and Cascade (Saito et al. 2021), involving just the larger TniQ protein (the shorter one participates in RNA-guided integration).

CASTs harbouring spacers that target iMGEs could represent a way to bolster gene transfer through crRNA-guided transposition, as opposed to the canonical CRISPR-Cas systems acting as genetic barriers. Once in the recipient cell, homing transposition would facilitate CAST integration into the host genome. However, the limited length of the CASTs CRISPR arrays and the fact that adaptation modules are invariably missing suggest that the expansion of the transposition sites repertoire is restricted. Still, spacers could be integrated de novo by compatible adaptation complexes encoded by CRISPR-Cas systems co-occurring in the cell.

## CRISPR/Cas in prokaryotic viruses

Deep in silico analyses have revealed the presence of CRISPR-Cas components in plasmids, viruses and proviruses. Complete CRISPR-Cas systems seem to be very infrequent in viral sequences. Moreover, even though many systems found in chromosomes have been tentatively assigned to provirus regions, CRISPR-Cas components could have been inserted within the viral region after provirus integration. Thus, just two reliable cases of complete CRISPR-Cas systems have been reported so far in viruses: the I-B system of *Clostridium botulinum* phage D-1873 and the I-F system of *Vibrio* spp. phages (Seed et al. 2013; Faure et al. 2019b). So far, only the latter has been experimentally validated. Interestingly, on the basis that carried spacers target a host anti-phage island, it has been proposed that the system in *Vibrio* phages might function as a counter-defence mechanism (Seed et al. 2013; Naser et al. 2017).

In viruses and proviruses, stand-alone CRISPR arrays and sequences like canonical repeat units (named solitary repeat units or SRUs) are significantly more common than complete CRISPR-Cas. SRUs resembling the CRISPR sequences in the host have been tentatively related to anti-CRISPR mechanisms (Faure et al. 2019b). According to the proposal, SRUs might act as dominant-negative inhibitors of the CRISPR-Cas machinery of the host by competing with bona fide crRNAs for binding to the effector proteins. It has also been envisaged that the integration of the viral genome within a host CRISPR-*cas* locus might occur via homologous recombination between SRUs and similar repeats in resident CRISPR arrays, interrupting their transcription.

Strikingly, some of the larger orphan arrays found in prokaryotic viruses contain spacers that match host genes or intergenic regions, suggesting that they may play regulatory roles (Al-Shayeb et al. 2020). However, most of these CRISPR arrays are very small (mini-CRISPR arrays), composed of a single spacer flanked by either two complete repeats or a complete repeat and a truncated CRISPR-like sequence. The sequences of these repeats in mini-arrays are in most cases identical or very similar to repeats of complete CRISPR-Cas systems present in the respective host genomes (Faure et al. 2019b; Medvedeva et al. 2019). Regarding the spacers, in contrast to the low percentage (roughly 10%) of spacers in prokaryotic genomes that match known sequences, it has been estimated that 67% and 93% of the spacers carried in mini arrays of viruses and proviruses, respectively, have a potential target (Shmakov et al. 2017b). Notably, most of these spacers match sequences in similar viruses or proviruses but not in the respective viral or host genome. Moreover, putative promoters have been tentatively identified upstream most mini arrays detected, suggesting that they are transcribed (Faure et al. 2019b). The absence of *cas* genes and other CRISPR sequences necessary for interference (e.g. tracrRNA genes are absent in the case of type II mini arrays) implies that the activity of these mini arrays depends on host CRISPR and Cas elements (Faure et al. 2019b; Medvedeva et al. 2019; Iranzo et al. 2020). These observations led to the hypothesis that solitary mini-CRISPR arrays hijack the host CRISPR-Cas systems to tackle virus superinfection. Thus, when a virus carrying a mini array infects the cell, crRNAs produced from the array would guide the host Cas effector proteins to inhibit infection by a targeted competitor virus.

Moreover, the viral mini array could expand its spacer repertoire acquiring new spacers from the second infecting virus through the adaptation machinery of the host. Superinfection inhibition and acquisition of new spacers have been experimentally demonstrated for mini arrays of viruses infecting the archaeon *Saccharolobus* spp*.* (Medvedeva et al. 2019)*.*

Finally, a mathematical model on the cost of mini-CRISPR array maintenance and productivity of co-infection events predicted that mini arrays should be more frequent in viruses with a narrower host range, where competition with co-infecting viruses is of prime relevance (Iranzo et al. 2020). According to the theoretical prediction, the mini-CRISPR arrays of viruses that infect the same cell might undergo a rapid co-evolution. As a result, each would be forced to update its spacers pool to re-enlist effective targeting against the respective competitor viruses when CRISPR-evading mutations arise.

Experimental confirmation of these theoretical concepts and the underlying mechanism involved in mini arrays functioning will allow understanding of their role and decipher their regulation, efficiency, and consequences within the microbial population.

## CRISPR-Cas control

Having CRISPR-Cas constantly turned on would allow for rapid neutralisation of iMGEs; however, uncontrolled expression may also have several disadvantages. The potential toxic effects of CRISPR-Cas action, notably that of the Cas nucleases acting on self-nucleic acids, and the fitness cost associated with the CRISPR-Cas expression involves the need for checkpoints of the CRISPR mechanism and regulatory strategies to fine-tune repression, induction, activation and inactivation at transcriptional, translational and post-translational levels (Patterson et al. 2017; Leon et al. 2018). Moreover, iMGEs have evolved diverse mechanisms to evade CRISPR-based interference. These aspects of CRISPR-Cas control are summarised in the following sections.

### Checkpoints of the CRISPR-Cas mechanism

Determinants of CRISPR-Cas functioning contribute to preventing cell toxicity. Concerning the first stage of CRISPR-Cas immunity, the self-targeting rate is reduced through the preferential uptake of spacers from foreign genetic elements during naïve adaptation (Levy et al. 2015), while primed acquisition leads to a biased integration of spacers derived from pre-targeted regions (Vorontsova et al. 2015). The adaptation complex can capture debris left by DNA repair machinery. In Gram-negative bacteria, upon recognising free DNA ends (Ivančić-Bace et al. 2015; Levy et al. 2015; Radovčić et al. 2018), RecBCD proceeds by degrading DNA until reaching a Chi site (Smith 2012). The AddAB system, a paralog of the RecBCD complex in Gram-positive bacteria, is necessary for efficient spacer acquisition in some of these microorganisms (Modell et al. 2017). Moreover, regions between free DNA ends and Chi sites are more prone to be acquired by the II-A system in *Streptococcus pyogenes* (Modell et al. 2017). The higher content of Chi sequences in the bacterial chromosome compared to the low frequency of Chi-like sequences usually found in transmissible genetic elements results in fewer spacer-donor regions in self-DNA than in foreign molecules, explaining in part the apparent preference for naïve acquisition of spacers derived from the latter (Levy et al. 2015). Although other repair proteins such as PriA and RecG are involved in primed adaptation in *E. coli* (Ivančić-Bace et al. 2015; Killelea and Bolt 2017; Radovčić et al. 2018), the precise role played and potential checkpoints remain to be elucidated.

Regarding the crRNA biogenesis stage, Cas ribonuclease-mediated cleavage of RNAs other than pre-crRNAs is impeded due to the recognition of specific sequences and either the stem-loop adopted by palindromic repeats or the structure formed by tracrRNA:pre-crRNA hybrids (see section above on ‘crRNA biogenesis’).

Cleavage by Cas interference nucleases requires the navigation of multiple checkpoints involving sequential conformational rearrangements of the effector protein that occur after binding to the guide RNA, the target or other Cas proteins (reviewed in Jackson et al. 2017). Thus, their nucleolytic capability is only activated during the recognition of guide-complementary targets (Sternberg et al. 2014; Hochstrasser et al. 2014).

Still, the activity of Cas nuclease effector proteins poses a risk to the cell if guided with crRNA targeting sequences that match resident regions. Targeting of the CRISPR locus by Cas nuclease effectors is prevented due to the requirement for a PAM (Westra et al. 2013a; Foster et al. 2020) or mismatches between the target and the crRNA beyond the spacer region (Marraffini and Sontheimer 2010; Meeske and Marraffini 2018; Foster et al. 2020). Finally, the collateral random ssDNA degradation and non-complementary RNA cleavage exhibited by type III systems only occur after binding of the surveillance complex to targeted RNA, thus limiting potential damage of the own nucleic acids to actively transcribed genetic elements (Samai et al. 2015; Jia et al. 2019; Sofos et al. 2020; Foster et al. 2020). Moreover, autoimmunity against the CRISPR locus that might be triggered by anti-sense transcripts generated from some type III CRISPR arrays is prevented by inhibition of Cas10 activities relying on base-pairing between target RNA and crRNA positions flanking the spacer region (Foster et al. 2020; Liu and Doudna 2020).

Indirect parameters such as DNA topology also have an impact on several steps of CRISPR-Cas activity (Westra et al. 2012a, b). For example, R-loop formation after hybridisation between the crRNA and the target dsDNA is influenced by the level of DNA negative supercoiling. Furthermore, DNA bending by hosts factors is required to facilitate recognition of the spacer integration site by the adaptation complex (Dorman and Ní Bhriain 2020).

### Regulation of CRISPR-*cas* expression and Cas activity by cellular regulatory networks

Although constitutive transcription from CRISPR and *cas* promoters has been observed in diverse systems (Mojica et al. 1993; Hale et al. 2008; Lillestøl et al. 2009; Juranek et al. 2012; Crawley et al. 2018), there is also evidence that transcription from some of these promoters is usually repressed. Typically, these regulated loci are expressed only in certain circumstances, notably when the cell is invaded by potential targets, under stress conditions or when the risk of infection is high (Agari et al. 2010; Quax et al. 2013a; Fusco et al. 2015; León-Sobrino et al. 2016; Patterson et al. 2016; Høyland-Kroghsbo et al. 2017; Yang et al. 2020).

Diverse regulatory proteins and RNAs affecting transcription of CRISPR-Cas components have been identified in bacteria and archaea (reviewed in Patterson et al. 2017). As a reflection of the divergent evolutionary paths on the regulation of CRISPR-Cas expression that each prokaryote can adopt, a given regulatory factor (i.e. cyclic AMP receptor protein) may either repress or activate promoters of homologous *cas* genes depending on the microorganism (Shinkai et al. 2007; Yang et al. 2014). Moreover, signalling mechanisms (e.g. cellular metabolic sensors, stress-responsive two-component systems, quorum sensing) are involved in CRISPR-Cas regulation. For example, it was shown that quorum sensing signals activate *cas* gene expression in I-E, I-F and III-A CRISPR-Cas systems of *P. aeruginosa* and *Serratia* species (Patterson et al. 2016; Høyland-Kroghsbo et al. 2017). Conceivably, many other bacteria might use this strategy to induce CRISPR immunity at high cell density when the risk of infection increases.

Furthermore, the translation efficiency of *cas* mRNAs, mainly determined by codon usage biases, has also been related to CRISPR-Cas effective functioning (Quax et al. 2013b), and a CRISPR repeat-binding protein was shown to facilitate transcription of a CRISPR locus (Deng et al. 2012). CRISPR-Cas interference can also be stimulated by Cas protein stabilisation (Yosef et al. 2011), and suppression of both interference and adaptation by *cas* mRNA-binding regulatory proteins has recently been reported for several CRISPR-Cas types (Campa et al. 2021).

The I-E systems from *E. coli* and *Salmonella enterica* strains have been studied in detail, providing an overview of the complexity that the regulation of CRISPR-*cas* loci may require, involving multiple, complementary and alternative dose-dependent factors. The two systems are tightly regulated at the transcription level by an elaborate regulatory network that involves several transcription factors and nucleoid-associated proteins. Besides, anti-sense RNAs detected in the *cas* loci of the two species might also be implicated in the regulation of Cas expression. In *E. coli*, Cas3 protein is stabilised by a chaperon protein induced upon phage infection (Yosef et al. 2011) and the histone-like nucleoid-structuring protein H-NS represses transcription from all *cas* and CRISPR promoters (Pul et al. 2010; Pougach et al. 2010). As H-NS-mediated silencing is achieved by its cooperative spreading on the promoter regions, DNA topology can have an impact on the activity of the system (Liu et al. 2010). Moreover, transcription from divergent promoters located in one of the intergenic regions of the I-E *cas* locus of *E. coli*, where H-NS binds (Pul et al. 2010), is expected to generate a local domain of high negative supercoiling (Mojica and Higgins 1996), therefore facilitating H-NS association and subsequent transcription inhibition. Related to this, H-NS-mediated gene silencing is frequently linked to changes in DNA secondary structure (Mojica and Higgins 1997; Winardhi et al. 2015).

Overexpression of the H-NS antagonist LeuO, a LysR-Type regulator, relieves repression of the I-E Cascade operon in *E. coli* (Mojica and Díez-Villaseñor 2010; Westra et al. 2010). Based on the preferential binding of H-NS to AT-rich DNA, it has been proposed that H-NS silencing of the *cas* loci could also be mitigated (Pul et al. 2010; Westra et al. 2010) upon infection by viruses or plasmids with high A-T content (Rocha and Danchin 2002) which would sequester part of the H-NS pool (Doyle et al. 2007; Dillon et al. 2010). The global regulator CRP (cAMP receptor protein) competes with LeuO for binding to the Cascade promoter, preventing LeuO-mediated activation (Yang et al. 2014). However, both strong activation and no significant effect of CRP on the *cas3* promoter have been reported, which has tentatively been related to the different growth phases of the *E. coli* cultures assayed in the two studies (Yang et al. 2014, 2020). Similarly, two recent publications (Mitić et al. 2020; Sun et al. 2020a) documented contradictory results showing that when the gene encoding the H-NS paralog StpA was inactivated or deleted in distinct genetic backgrounds, the *cas* operon transcription was either increased (in an *hns cas1* double mutant) or reduced (*hns* null mutant). Nevertheless, overexpression of StpA suppressed transcription in both cases. These inconsistencies could be due to the experimental specificities of each study. Meanwhile, in addition to silencing by H-NS and positive regulation by LeuO, transcription of the CRISPR-Cas system from *S. enterica* serovar Typhi is repressed by the leucine-responsive regulatory protein LRP, and, also in contrast with *E. coli*, CRP does not participate in its transcriptional control (Medina-Aparicio et al. 2011, 2017).

### CRISPR-Cas self-control

There are appealing examples of CRISPR-Cas control executed by canonical Cas and CRISPR arrays, as well as by transcriptional regulators associated with these loci.

In addition to the I-E system, many *E. coli* strains harbour components of an I-F CRISPR-Cas system (Díez-Villaseñor et al. 2010). However, most evolutionary lineages of *E. coli* have lost all I-F *cas* genes and only a small CRISPR array remains. Interestingly, the best matches with the spacers of these orphan arrays correspond almost invariably to sequences of I-F *cas* genes found in related strains. This observation suggested that the acquisition of *cas*-targeting spacers might have been responsible for the loss of these genes. Accordingly, it was shown that native orphan arrays can elicit interference against plasmids carrying a complete set of I-F *cas* genes (Almendros et al. 2016). Therefore, it was proposed that Cas proteins were guided by the constitutively expressed orphan arrays against the targeted *cas* genes. Moreover, this targeting resulted in the primed acquisition of plasmid-derived spacers that further boosted the degradation of the plasmid. Thus, these orphan arrays behave as a natural anti-*cas* mechanism that efficiently prevents the establishment in the cell of a cognate CRISPR-Cas system and promotes the destruction of the carrier genetic element.

Within the framework of CRISPR-related regulatory proteins, it has been recently shown that the MM_0565 protein associated with the subtype I-B CRISPR-*cas* locus of *Methanosarcina mazei* Gö1 binds to the leader of its I-B CRISPR array and a similar leader region in another CRISPR-Cas system (III-C subtype) present in the genome (Ulbricht et al. 2020). However, expression of the CRISPR-*cas* loci is not affected by overexpression of this protein, suggesting that it might play a post-transcriptional function influencing spacer integration through direct recruitment of the adaptation complex or affecting the structure of the leader, similarly to the role played by IHF regulatory protein in the I-E CRISPR-Cas system from *E. coli* (Nuñez et al. 2016).

The I-C system of *Myxococcus xanthus* offers another example of autoregulation. Its *cas* locus encodes eight proteins, including DevT (Cas8c), DevR (Cas7) and DevS (Cas5). It has been proposed that DevTRS form a Cascade-like subcomplex that negatively autoregulates the *dev* transcript (Rajagopalan and Kroos 2017). Even though the mechanism of transcription regulation by DevTRS is unknown, the canonical CRISPR-Cas interference, based on crRNA-guided target-cleavage, is not involved as CRISPR spacers matching the target seem to be absent, and activity of Cas nucleases (Cas3 and Cas6) is not required for this feedback regulation. However, neither non-crRNA guiding nor recruitment of non-Cas nucleases to the RNA or DNA target can be ruled out.

Likewise, a recent work (Workman et al. 2021) demonstrated self-regulation of a CRISPR-Cas system from *S. pyogenes*. In some subtype II-A systems, an extended tracrRNA (tracr-L) not found in other CRISPR-Cas subtypes is transcribed upstream of the tracrRNA promoter (Deltcheva et al. 2011). Workman and colleagues showed that the tracr-L of *S. pyogenes* guides Cas9 to repress the promoter of its *cas* operon. Downstream of this *cas* promoter, adjacent to the canonical 2-nt PAM of the CRISPR-Cas system, there is an 11-nt sequence complementary to the 5′ end of tracr-L. Such a short match still grants efficient binding of the Cas9:tracr-L repressor complex to the target site within the *cas* promoter. However, target cleavage appears to be prevented (Workman et al. 2021).

On the contrary, it has been reported that the subtype I-A associated protein Csa3a from *S. islandicus* activates the expression of its CRISPR adaptation module (Liu et al. 2015, 2017; León-Sobrino et al. 2016). The Csa3b protein encoded in the same locus, on the one hand, represses expression of the I-A interference genes (He et al. 2017), whereas on the other acts as a transcriptional activator of the two III-B effector complex gene cassettes present in the genome (Ye et al. 2020).

### How do invasive mobile genetic elements escape from CRISPR-Cas immunity?

Prokaryotic iMGEs have developed diverse strategies to evade CRISPR-Cas, which affect almost every CRISPR-Cas component (recently reviewed in Malone et al. 2021).

Viruses frequently escape CRISPR-defence after deletions (Pyenson et al. 2017; Watson et al. 2019), rearrangements or point mutations in the regions of their genome targeted by CRISPR spacers (Andersson and Banfield 2008). These changes may result in partial or complete removal of the target, mismatches between the crRNA and the target or disruption of the PAM recognised by the effector Cas proteins. Consequently, although effective cleavage may be temporarily avoided, these point mutations could still prompt primed spacer acquisition by some CRISPR-Cas systems, reassembling effective immunity (Semenova et al. 2011; Fineran et al. 2014; Jackson et al. 2019).

Otherwise, DNA chemical modifications of the viral genome can hamper the recognition of sequences targeted by the CRISPR-Cas machinery, although this has only been shown for one phage and host system (Bryson et al. 2015; Vlot et al. 2018).

Viral proteins account for other strategies of counter-defence. Some plasmids and viruses carry genes encoding H-NS homologues (Skennerton et al. 2011; Shintani et al. 2015). It has been proposed that these proteins might repress CRISPR-*cas* transcription as H-NS and StpA do in the I-E systems of enterobacteria (Dorman and Ní Bhriain 2020). Similarly, it is conceivable that genetic elements targeted by CRISPR-Cas systems relying on activation by quorum sensing could have deployed quorum quenching strategies (Mion et al. 2019). Actually, it has been recently demonstrated that a *P. aeruginosa* phage encodes a lactonase protein that disrupts the communication pathway responsible for the activation of the CRISPR-Cas systems in the host by inhibiting the receptor of the autoinducer molecule (Shah et al. 2021). In addition, manipulation of the host bacterial quorum sensing pathway has been reported for other viruses (Duddy and Bassleri 2021), suggesting that this might be a procedure frequently used by viruses for CRISPR evasion.

Bondy-Denomy and coworkers discovered in 2013 a CRISPR-Cas evasion strategy based on the so-called anti-CRISPR (Acr) proteins (Bondy-Denomy et al. 2013). Acr encoding genes found in prophages of *P. aeruginosa* were able to counteract the immunity provided by the host I-F CRISPR-Cas system. Since then, the collection of Acr proteins identified has widely extended, as has an interest in this line of research, due to the potential of Acrs in various CRISPR-Cas technologies (Liu et al. 2020b).

Acr proteins suppress CRISPR-Cas immunity mainly through two mechanisms: obstruction of DNA binding or inhibition of target cleavage (i.e. the surveillance complex binds to the DNA target site, but cleavage is not achieved) (Pawluk et al. 2014; Yang and Patel 2017; Marino et al. 2018; Watters et al. 2018; Knott et al. 2019b; Liu et al. 2019b; Bhoobalan-Chitty et al. 2019; Yin et al. 2019; Lin et al. 2020) In type I CRISPR-Cas systems, some Acrs block target cleavage by recruiting the effector nuclease Cas3 (Wang et al. 2016). Acr can also thwart target binding by type I surveillance complexes via direct interaction with Cascade or via DNA mimicry (i.e. Acr mimics dsDNA). In the first case, upon binding to Cascade, Acr triggers conformational changes that sterically occlude the DNA binding sites (Bondy-Denomy et al. 2015; Maxwell et al. 2016). In the second case, the Acr protein adopts a conformation resembling dsDNA and binds to the target site in the Cascade:crRNA complex instead of the target DNA (Guo et al. 2017; Peng et al. 2017). Acr proteins against type III systems have also been discovered that affect cOAs signalling (Peng et al. 2020; Athukoralage et al. 2020).

In class 2, most Acr proteins inhibit target binding to the surveillance complex using mechanisms equivalent to those exhibited by Acr acting against type I systems. For example, regarding the prevention of target cleavage, it has been observed that some Acrs bind catalytic residues of Cas9 blocking its nuclease activity (Pawluk et al. 2016a, b; Wang et al. 2016; Harrington et al. 2017). However, strategies, leading to (i) dimerisation of the effector protein (thus resulting in the reduction of dsDNA binding), (ii) cleavage of the crRNA spacer sequence in the surveillance complex and (iii) acetylation of specific residues of the Cas protein involved in PAM recognition, have been reported for type II and V systems as well (Marino et al. 2018; Dong et al. 2019; Knott et al. 2019a, b; Zhu et al. 2019). Although Acrs that inhibit the interference activity of type VI systems have been identified, the molecular mechanism involved remains to be elucidated (Smargon et al. 2017; Lin et al. 2020; Peng et al. 2020).

CRISPR and Cas elements encoded in viruses may represent another way to interfere with the host CRISPR-Cas activity (see the “CRISPR/Cas in prokaryotic viruses” section). For instance, regarding Cas proteins, many bacterial and archaeal viruses encode Cas4-like proteins that are thought to participate in counter-CRISPR mechanisms. Their proximity to Cas-inhibiting (i.e. *acr*) genes supports this hypothesis (Hooton and Connerton 2015; Hooton et al. 2016).

Lastly, Jumbo phages deploy a refined counter-CRISPR mechanism. During infection of the host bacterium, a peculiar nucleus-like structure is formed within which phage DNA replication and transcription takes place. Thus, its DNA is protected from degradation by CRISPR-Cas and restriction-modification systems (Modell et al. 2020; Malone et al. 2020).

## Conclusion

Invasive mobile genetic elements put intense selective pressure on bacteria and archaea. This challenge has resulted in numerous prokaryotic defence strategies, notably in the CRISPR-Cas adaptive immune systems. However, the fitness costs associated with CRISPR-Cas may result in stringent regulation of their expression, protein inactivation or, ultimately, loss of the complete system. In addition to immunity, many CRISPR-Cas systems play collateral or alternative roles. These side functions take advantage of the guiding capability of CRISPR RNAs and the multiple roles of Cas proteins in nucleic acid metabolism. Arguably, the associated benefits might account for the maintenance of the CRISPR-Cas systems even when foreign threats could be faced using less-costly protection mechanisms.

The discovery of the innate immunity provided by restriction-modification systems led to the birth of genetic engineering, allowing the cutting and pasting of DNA fragments in vitro almost at will. At present, the versatility of CRISPR-Cas adaptive immune systems has been translated into an arsenal of molecular biology tools that have revolutionised biotechnology, medicine and agriculture. Unfortunately, the research and understanding of the CRISPR-Cas systems that made this scientific revolution possible are often overlooked due to the more attention-grabbing advances in biotechnology and medical applications. Nevertheless, as we have described above, CRISPR-Cas biology is no less impressive. On the contrary, more than three decades after the discovery of the enigmatic repeats in prokaryotes, CRISPR research continues to shed light on the intricacies and complexity of this fascinating system.

## Data Availability

Not applicable.
